# Effects of ebselen and N-acetyl cysteine on replicative aging of primary human fibroblast strains

**DOI:** 10.1186/s12979-015-0035-y

**Published:** 2015-07-16

**Authors:** Shiva Marthandan

**Affiliations:** Leibniz Institute for Age Research, Fritz Lipmann Institute e.V. (FLI), Jena, Germany

## Abstract

Administration of selected concentrations of ebselen and N-acetyl cysteine have been proven to display an antioxidant potential based on their effect on markers of T cell integrity and function in human peripheral blood mononuclear cells and CD4^+^ T cell clones. Here we assessed the impact of various antioxidant concentrations on replicative aging of primary human fibroblast strains derived from embryonic lung (MRC-5) and foreskin (HFF). None of the antioxidant concentrations affected the cumulative population doublings, levels of oxidative DNA damage, intracellular GSH:GSSG ratio, potency of heat shock responses and the induction of senescence in both fibroblast strains. Our results showed no effect of both antioxidants on primary fibroblast strains and reveal their cell type specific antioxidant potential.

**Electronic supplementary material:**

The online version of this article (doi:10.1186/s12979-015-0035-y) contains supplementary material, which is available to authorized users.

## Introduction

Ebselen is a synthetic seleno-organic reactive oxygen species (ROS) scavenging compound mimicking glutathione peroxidase, an antioxidant selenoenzyme capable of reducing hydrogen peroxide (H_2_O_2_) [1]. N-acetyl L-cysteine (NAC), a precursor of glutathione (GSH) is another antioxidant involved in cellular detoxification [2]. A previous study revealed the antioxidant potential of both ebselen and NAC, respectively, in human peripheral blood mononuclear cells and CD4^+^ T cells derived from healthy young (25–30 year olds) and older aged (55–60 year olds) donors. We observed significantly higher intracellular GSH:GSSG ratio, reduced levels of oxidative DNA damage and increased proliferative capacity in either of the antioxidant treated samples [3]. A similar scenario with addition to increase in cumulative population doublings (PDs) were observed in human peripheral blood derived CD4^+^ T cell clones (TCCs) from healthy 26 year and 45 year old donors [3].

In this study, we investigated the effect of a range of concentrations of ebselen or NAC on replicative aging of primary human fibroblasts and studied whether these compounds had an impact on their transition into senescence. Previous studies in primates have revealed an age-dependent increase in the percentage of senescent skin fibroblasts *in vivo*, justifying the use of fibroblast strains as a model system in our senescence study [4]. Human fibroblasts undergo a certain number of population doublings (PDs) varying from 50 to 80, depending on fibroblast strain type, before reaching a state of permanent cell cycle arrest. This arrest makes them an ideal model system for investigating senescence [5]. Cellular senescence is a state where cells are metabolically active. Induction of senescence results in a decline in tissue integrity and function, ultimately resulting in a number of aging-associated diseases [6]. Many mechanisms and pathways (mainly the p53-p21 and p16-pRB pathways) have been proved to be responsible for cellular senescence [7]. Though there is no single marker that can characterize this state, mainly the increase in percentage of senescence associated β Galactosidase (SA - β Gal) staining has been pre-dominantly used to distinguish the senescent state [8]. Cellular senescence of some types of primary mammalian cells in culture mirrors *in vivo* aging mechanisms [9]. The aim of this investigation was to determine the impact of ebselen or NAC supplementation on replicative aging *in vitro*, in primary human fibroblasts.

### Cell type specific effects of selected concentrations of ebselen and NAC

Primary human fibroblasts MRC-5 (*Homo sapiens*, 14 weeks gestation male, fibroblasts from normal lung, ATCC) [10] and human foreskin fibroblasts (HFF, *Homo sapiens*, fibroblasts from foreskin) [11] were maintained in culture as described [12] and were supplemented with a range of concentrations of either ebselen (0, 15, 30, 60, 100 μM) or NAC (0, 2.5, 5, 7.5, 10 mM). Fibroblast culturing medium containing different antioxidant concentrations was changed twice per week, mimicking the culture conditions subjected to TCCs and human peripheral blood in our previous studies [3, 13]. Both of these compounds have been known for their antioxidant potential in a number of other cell types [3, 13]. However, we observed no significant change in the cumulative PDs in both fibroblast strains, irrespective of their concentrations. Investigation on the induction of senescence measured by SA - β Gal assay, intracellular GSH:GSSG ratio (Additional file 1: Table S1) and levels of oxidative DNA damage (Additional file 1: Tables S2 and S3) determined by the GSH:GSSG ratio assay kit and modified alkaline comet assay respectively [3, 13] revealed no significant changes on either of the antioxidant supplementations compared to the control strains.

We also determined the effect of either of the antioxidant (30 μM ebselen or 7.5 mM NAC) on the levels of phosphorylation of HSP27, an important member in the family of heat shock proteins. Neither of the antioxidant treatments had an effect on the increase in HSP27 phosphorylation with age in either MRC-5 (Additional file 1: Figure S1) or HFF strains. The increase in levels of HSP27 in response to aging in skin fibroblasts has been observed previously [14, 15]. Thus the antioxidant treatments did not interfere with the gradual decline in heat shock responses with age which ultimately contributes to the increase of protein damage [16]. Furthermore, preliminary findings show no significant impact of either of the antioxidants on the induction of cyclin dependent kinase inhibitors p21 and p16 with age. The protein levels of HSP27, p21 and p16 were determined by immunoblotting in the antioxidant-treated strains compared to the controls.

Though our previous study revealed the ROS scavenging potential of selected concentrations of ebselen (30 μM) and NAC (7.5 mM) demonstrated by their impact on several markers of T cell integrity and function, higher concentrations of ebselen (60 – 100 μM) or NAC (10 mM) resulted in inhibition of the growth of TCCs. In terms of fibroblasts, higher concentrations of both antioxidants did not demonstrate a significant effect on the growth of fibroblast cell strains used in this study. The cumulative PDs remained unchanged. The possible explanation for the lack of hormetic (beneficial) effect of any of the antioxidant concentrations may be due to the fact that existing levels of background biomolecule damage in primary human fibroblasts may be enough to negate the ROS scavenging potential of these specific concentrations of ebselen or NAC. A lower or a relatively higher concentration of either of the compounds may reveal a ROS scavenging capacity in primary human fibroblasts. However, much higher concentrations of 200 μM ebselen or 30 mM NAC resulted in the inhibition of growth of fibroblast strains independent of their cell origin. Interestingly in a previous study, 10 mM NAC administration attenuated γ-H_2_AX and pATM (Ser-1981) formation in MRC-5 fibroblasts maintained in conditioned media [17].

Healthy aging being attributed to preservation of cellular homeostasis in response to stress thanks to the effective functioning of a complex network of processes controlled by vitagenes encoding heat shock proteins, thioredoxins and the sirtuin protein systems has been extensively documented [18, 19]. Dietary antioxidants have been demonstrated to activate pathways including vitagenes [19]. This concept could be responsible for the antioxidant potential of ebselen or NAC in human T cells when supplemented from an early age during their in vitro lifespan [3]. Our overall findings here show no significant effect of any of the concentrations of either of the antioxidants on replicative aging of human fibroblast strains (Table 1).

**Table 1:** Effect of 30 μM ebselen or 7.5 mM NAC administration in different primary human cell types compared to controls

Human CD4^+^ TCCs	Human CD4^+^ TCCs	Human peripheral blood & CD4^+^ T cells *ex vivo* (derived from 25–30 or 55–60 year old donors) [3]	MRC-5 (Human embryonic lung fibroblasts)	HFF (Human foreskin fibroblasts)
26 & 45 year old donors [3]	80 year old donors [13]			
Increase in cumulative PD*	No effect on cumulative PD	Increase in proliferation capacity*	No effect on cumulative PD	
			No effect on induction of SA – β Gal	
Increase in intracellular GSH:GSSG ratio*	No effect on intracellular GSH:GSSG ratio	Increase in intracellular GSH:GSSG ratio*	No effect on induction of cyclin dependent kinase inhibitors (p16, p21)	
Decrease in levels of oxidative DNA damage*	No effect in levels of oxidative DNA damage	Decrease in levels of oxidative DNA damage*	Intracellular GSH:GSSG ratio remains unchanged	
			No significant changes on the levels of oxidative DNA damage	
No effect on intracellular total glutathione levels	No effect on intracellular total glutathione levels			
		Increase in intracellular total glutathione levels*	No effect on the increase in phosphorylation of HSP27 with age	

The effects of 5 mM NAC administration were similar to 7.5 mM NAC in human peripheral blood and CD^+^ T cells *ex vivo* irrespective of the age of the donors. 10 μM ebselen or 1.25 mM NAC administration did not have any impact on any of the markers investigated compared to controls in all the cell types and strains. 60 and 100 μM ebselen or 10 mM NAC resulted in inhibition of growth of all the three cell types investigated (Human CD4^+^ TCCs, peripheral blood mononuclear cells and CD4^+^ T cells *ex vivo*) whereas these concentrations of antioxidants did not have an effect on either of the cell strains (MRC-5 and HFF) compared to controls. All the primary human cell types and strains were maintained in culture in presence of either of the antioxidants throughout their span in culture. *p < 0.05 compared to controls

## Conclusion

This study reveals the cell type specific antioxidant potential of selected concentrations of ebselen and NAC. Though we previously demonstrated the ROS scavenging capabilities of specific concentrations of either of the antioxidants in human peripheral blood mononuclear cells and TCCs, their administration in culture does not have an effect on the replicative aging of primary human fibroblasts as confirmed by the lack of impact on a number of markers investigated in this study.

## Additional file

Additional file 1: Table S1. Levels of oxidative DNA damage in primary human fibroblast strains on antioxidant supplementation. **Table S2.** GSH:GSSG ratio of MRC-5 fibroblast strains on antioxidant supplementation. **Table S3.** GSH:GSSG ratio of HFF strains on antioxidant supplementation. **Figure S4.** Impact of antioxidant supplementation (30 μM ebselen or 7.5 mM NAC) on HSP27 phosphorylation in MRC-5 fibroblast strains.
