# Supplementary material for: Effects of ebselen and N-acetyl cysteine on replicative aging of primary human fibroblast strains
Source: Immun Ageing. 2015 Jul 16;12:8. doi: 10.1186/s12979-015-0035-y (PMC4502570; doi:10.1186/s12979-015-0035-y)

**Supplement Section**

**Table S1. Levels of oxidative DNA damage in primary human fibroblast strains on antioxidant supplementation**

The levels of oxidative DNA damage in primary human fibroblast strains (MRC-5 and HFF) at different PDs supplemented + / - 30 μM ebselen or 7.5 mM NAC (n=3)

| **Fibroblast →** | | **MRC-5** | | | **HFF** | | |
| --- | --- | --- | --- | --- | --- | --- | --- |
| **PD →** | | **34** | **50** | **70** | **20** | **48** | **72** |
| **30μM ebselen** | **-** | 47.8±1.5 | 36.5±2.1 | 24.0±3.5 | 46.3±3.4 | 36.2±0.5 | 18.7±0.9 |
|  | **+** | 47.5±0.8 | 34.7±1.7 | 23.5±1.0 | 47.9±0.9 | 35.8±0.8 | 17.9±0.6 |
| **7.5mM**  **NAC** | **-** | 47.2±1.6 | 36.0±2.0 | 22.8±0.3 | 49.9±2.6 | 38.3±0.6 | 23.7±2.2 |
|  | **+** | 45.2±0.7 | 35.4±1.0 | 22.1±0.6 | 49.9±0.7 | 37.5±0.4 | 22.2±0.8 |

**Table S2. GSH:GSSG ratio of MRC-5 fibroblast strains on antioxidant supplementation**

GSH:GSSG ratio of MRC-5 fibroblast strains at different PDs supplemented + / - 30 μM ebselen or 7.5 mM NAC (n=3)

| **Fibroblast →** | | **MRC-5** | | | | | | | | |
| --- | --- | --- | --- | --- | --- | --- | --- | --- | --- | --- |
| **PD →** | | **34** | | | **50** | | | **70** | | |
| **Comet assay** | | **Alk** | **End** | **Fpg** | **Alk** | **End** | **Fpg** | **Alk** | **End** | **Fpg** |
| **30μM**  **ebselen** | - | 20.2±2.2 | 21.5±2.5 | 21.9±1.4 | 33.7±1.2 | 34.9±1.3 | 35.1±1.2 | 47.2±0.8 | 49.1±0.7 | 51.9±1.5 |
|  | + | 20.9±1.6 | 22.5±0.5 | 23.1±0.7 | 35.2±0.8 | 36.8±0.9 | 36.7±1.0 | 48.5±1.3 | 50.8±1.8 | 52.3±1.7 |
| **7.5mM**  **NAC** | - | 23.2±0.6 | 24.4±0.4 | 23.9±2.2 | 34.9±0.5 | 35.7±0.5 | 36.7±0.6 | 46.9±1.6 | 47.3±1.3 | 48.9±1.8 |
|  | + | 22.4±0.9 | 23.5±0.5 | 23.4±0.4 | 35.6±0.4 | 36.4±0.9 | 36.3±0.3 | 47.9±1.7 | 49.2±1.3 | 50.8±1.1 |

**Table S3. GSH:GSSG ratio of HFF strains on antioxidant supplementation**

GSH:GSSG ratio of HFF strains at different PDs supplemented + / - 30 μM ebselen or 7.5 mM NAC (n=3)

| **Fibroblast →** | | **HFF** | | | | | | | | |
| --- | --- | --- | --- | --- | --- | --- | --- | --- | --- | --- |
| **PD →** | | **20** | | | **48** | | | **72** | | |
| **Comet assay** | | **Alk** | **End** | **Fpg** | **Alk** | **End** | **Fpg** | **Alk** | **End** | **Fpg** |
| **30μM**  **ebselen** | - | 19.8±0.5 | 20.7±1.0 | 21.2±1.1 | 33.0±0.6 | 34.0±0.2 | 34.4±0.3 | 50.3±1.0 | 51.0±1.3 | 51.3±1.5 |
|  | + | 20.3±1.1 | 21.7±1.5 | 22.4±1.0 | 35.5±0.8 | 36.2±0.8 | 36.2±0.3 | 51.3±1.0 | 52.2±1.6 | 52.5±1.3 |
| **7.5mM**  **NAC** | - | 23.7±0.6 | 23.9±0.6 | 24.5±0.8 | 34.4±1.1 | 35.3±0.6 | 35.4±0.9 | 48.6±1.1 | 49.4±0.4 | 49.8±0.3 |
|  | + | 23.4±1.0 | 24.7±0.7 | 24.4±0.4 | 35.3±1.0 | 36.0±1.4 | 36.7±1.5 | 49.0±1.5 | 49.2±1.4 | 49.7±1.2 |

**Figure S4. Impact of antioxidant supplementation (30μM ebselen or 7.5mM NAC) on HSP27 phosphorylation in MRC-5 fibroblast strains**


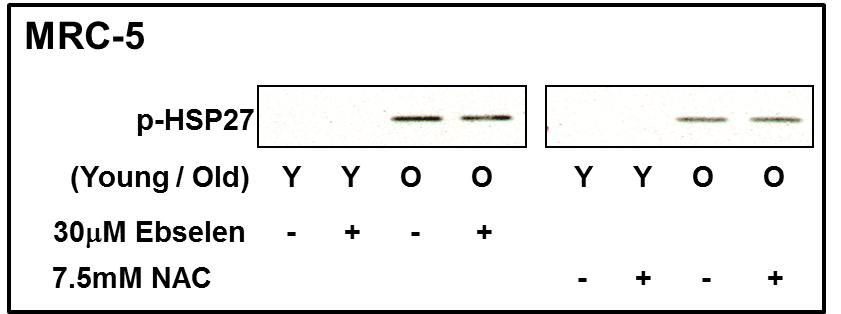

Supplement: Additional file 1: Table S1. — Levels of oxidative DNA damage in primary human fibroblast strains on antioxidant supplementation. Table S2. GSH:GSSG ratio of MRC-5 fibroblast strains on antioxidant supplementation. Table S3. GSH:GSSG ratio of HFF strains on antioxidant supplementation. Figure S4. Impact of antioxidant supplementation (30 μM ebselen or 7.5 mM NAC) on HSP27 phosphorylation in MRC-5 fibroblast strains. [file 12979_2015_35_MOESM1_ESM.docx]
